# Diffuse uveitis and chorioretinal changes after yellow fever vaccination: a re-emerging epidemic

**DOI:** 10.1186/s40942-019-0180-0

**Published:** 2019-10-07

**Authors:** Paula M. Marinho, Heloisa Nascimento, Andre Romano, Cristina Muccioli, Rubens Belfort

**Affiliations:** Department of Ophthalmology, Federal University of Sao Paulo, R. Botucatu, 822 - Vila Clementino, São Paulo, SP 04021-001 Brazil

**Keywords:** Yellow fever, Vaccination, Puntiform choriocapillaris, Uveitis

## Abstract

**Background:**

With increasing incidence of yellow fever, mass campaign vaccinations are underway and little ophthalmological alterations have been reported in literature, specially regarding non-combined vaccines.

**Case presentation:**

We report the case of a patient with no previous ocular or systemic diseases whom received a single dose of yellow fever vaccination and developed haematological, hepatic and renal alterations progressing with a later onset bilateral asymmetric diffuse uveitis. Ophthalmological findings included fine keratic precipitates scattered throughout the cornea and mild vitritis. Multimodal evaluation showed subtle puntiform choriocapillaris changes with decreased vascular density associated. The patient had a good visual outcome after mild oral prednisone dose, but the image findings have not presented remission.

**Conclusions:**

Clinicians should be aware of clinical and subclinical ocular manifestations such as subtle puntiform choriocapillaris changes as possible vaccine-related adverse events with potential to impact vision.

## Background

Yellow fever (YF) incidence rates are increasing in many countries and in large cities in Brazil, such as São Paulo and Rio de Janeiro, a significant amount of lethal cases are occuring since 2017 [1] leading to mass vaccination campaigns. Due to emerging demand, federal government used fractional dose (one-fifth of original dose) to amplify vaccination coverage. Although reported among the safest vaccines available [2] serious adverse events such as neurological complications and also vasculopathies have been reported, mainly after combined vaccination against YF and other infectious entities [3, 4] with few information about isolated YF vaccine available. It remains unknown the interaction between the host immune system and the vaccine leading to those events [2].

## Case presentation

A 50-year-old caucasian Brazilian woman with no previous ocular or systemic diseases received one dose of YF vaccine in April 2017 (Biomanguinhos/Fiocruz, lot 160VFA0433, Rio de Janeiro, Brazil). After 4 days, she presented with fever, hyporexia, and nausea. Laboratory studies showed leukopenia [3.340/mm^3^—normal range (NR): 3.500–11.500/mm^3^], thrombocytopenia (15.000/mm^3^—NR: 150.000–450.000/mm^3^), elevated C-reactive protein (33.34 mg/dL—NR: < 1.00 mg/dL), leukocyturia (80.000/mL—NR: < 30.000/mL), hematuria (29.000/mL—NR: < 12.000/mL), elevated creatinine (2.58 mg/dL—NR: 0.6–1.10 mg/dL), elevated urea (112 mg/dL—NR: 10–50 mg/dL), jaundice (bilirubin, 4.11 mg/dL—NR: 0.00–0.30 mg/dL), and moderately increased liver enzymes. The patient was admitted for intensive care with support measures and monitorization. Other infectious entities such as Cytomegalovirus, Herpes, Measles, Toxoplasmosis, Dengue Fever and Hepatitis A were discarded.

**Fig. 1:**
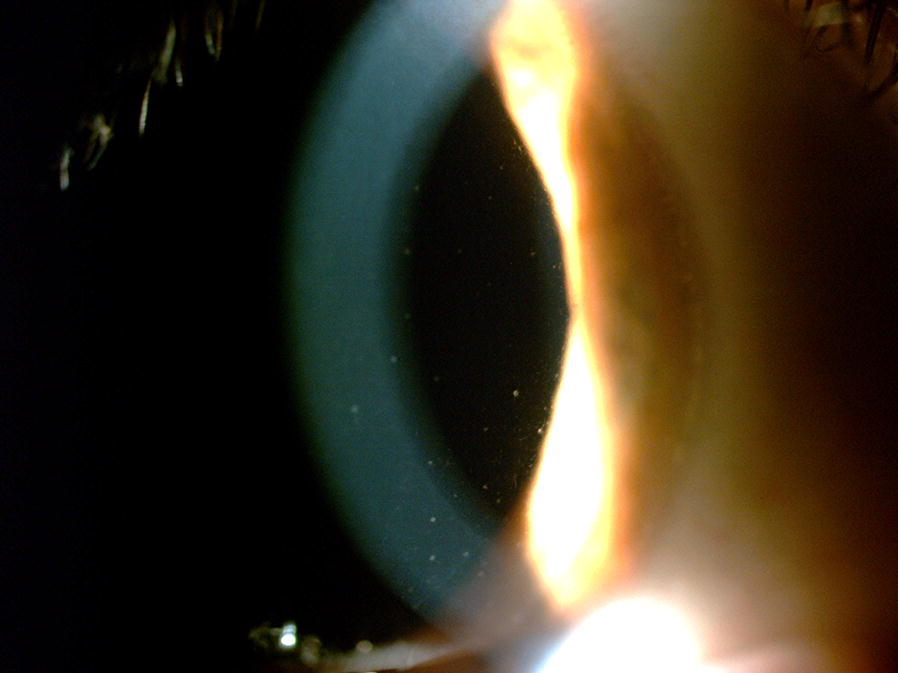
Anterior segment presenting fine diffuse keratic preciptates

**Fig. 2:**
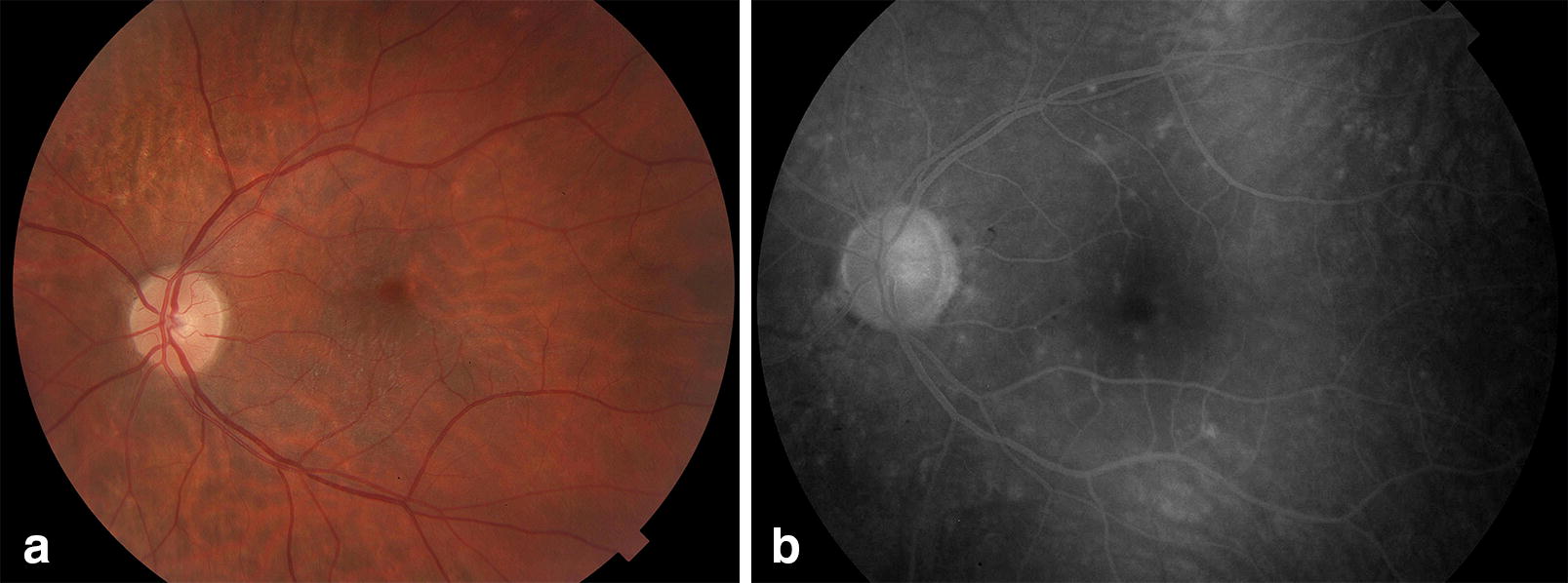
**a** Left eye retinography. **b** Late-stage fluorescein angiography showing focal hyperfluorescence dots at the posterior pole in the left eye

**Fig. 3:**
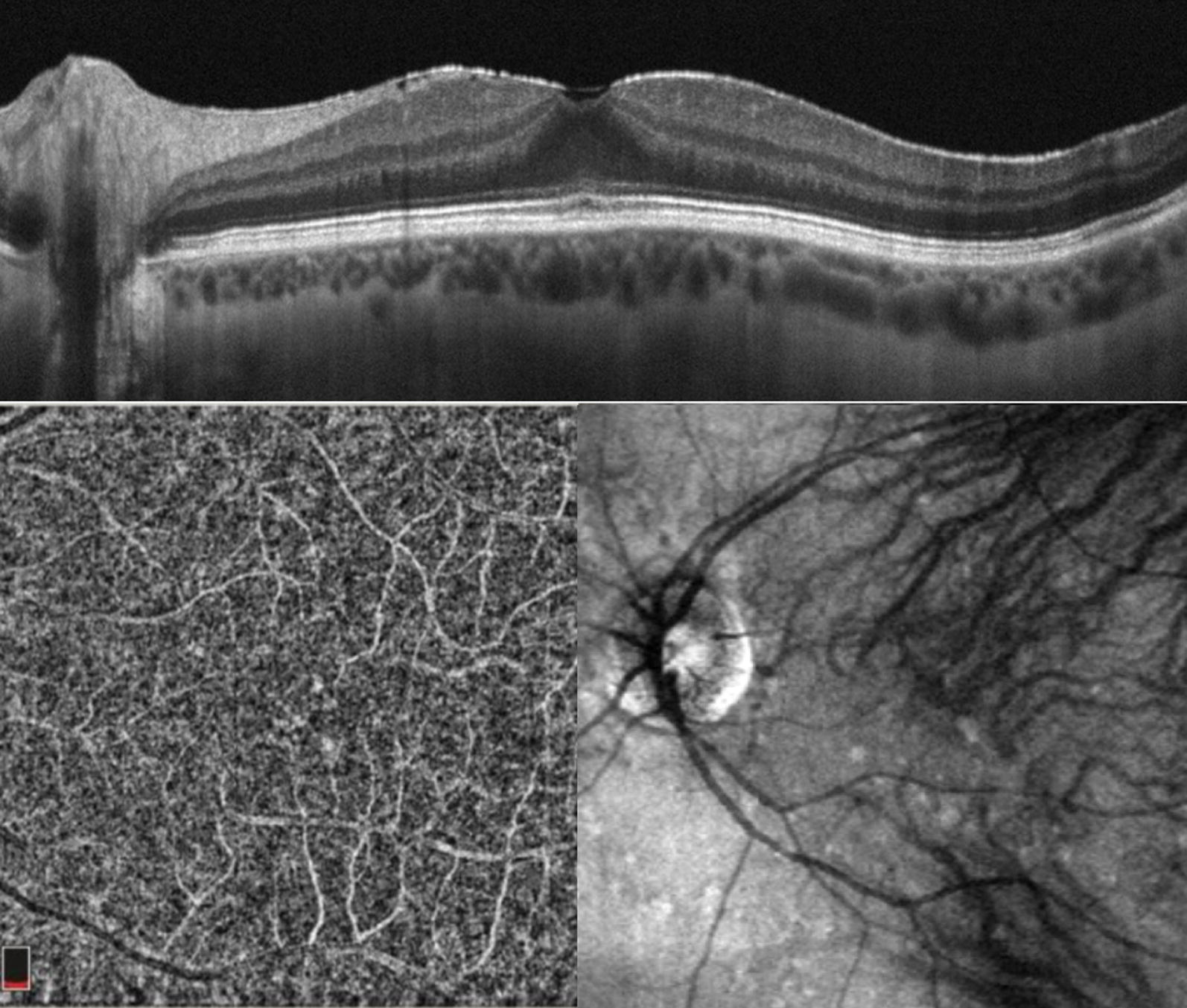
Cross-section optical coherence tomography (OCT) revealed an epiretinal membrane. Structural En Face OCT depicted diffuse hyporeflective dots at the choriocapillaries at the same location with OCT angiography

Approximately 12 days after symptoms onset, she reported blurred vision bilaterally. When ophthalmological evaluation was performed best-corrected visual acuity was 20/20 in right eye and 20/30 in left eye, and intraocular pressure was normal bilaterally. Examination showed fine keratic precipitates scattered throughout the cornea (Fig. 1) and mild vitritis bilaterally. Neither fundoscopy nor colored fundus photography have presented any relevant information but a discrete opacity of means in both eyes. Late-stage fluorescein angiography showed focal hyperfluorescence dots at the posterior pole in the left eye (Fig. 2); fundus autofluorescence disclosed widely dispersed hypoautofluorescent dots that were more prominent ipsilaterally. Cross-sectional optical coherence tomography (OCT) showed bilateral epiretinal membranes. Structural en-face OCT (Avanti, Optovue, Fremont, CA) shows diffuse hyporeflective dots at the level of the choriocapillaries with decrease vessel density at the same location with OCT angiography in the left eye (Fig. 3). Bilateral asymmetric diffuse uveitis was diagnosed and oral prednisone (40 mg/daily) was started. Patient was reevaluated after a week with same findings, and every two weeks after with progressive improvement. After 40 days inflammation signs disappeared, hence improving fundus imaging. Neither remission of the changes in the choriocapillaris nor vascular changes where seen during this period. Patient had no more visual complaints and visual acuity has improved to 20/20 OU.

## Discussion and conclusions

Only in Brazil, approximately 20 million people are expected to be vaccinated against YF and many reactions can occur [5]. Likewise the recent epidemics of Zika virus and Ebola virus associated uveitis [6], YF may have a polymorphic presentation.

We present a case of a rare adverse reaction to yellow fever vaccine in which a patient developed acute panuveitis. The possible etiologies of our patient’s reaction include a type IV hypersensitivity to the vaccine or its components. The yellow fever vaccine is known to contain live attenuated virus, gelatin, and sorbitol. A review of the literature identified multiple published reports of a gelatin hypersensitivity causing symptoms of anaphylaxis, but there has never been a published case report of a type IV hypersensitivity reaction to gelatin [7]. Sorbitol has also been implicated, albeit rarely, in type I hypersensitivity [8, 9].

Likewise our patient, Biancardi and Moraes [10] reported ocular inflammation cases after YF fractional dose vaccination but presenting as anterior or intermediate uveitis. Stangos et al. [11] as well as Escott et al. reported cases of posterior uveitis but secondary to combined vaccination for yellow fever and hepatitis A. All cases, similarly to our patient, had good outcomes with inflammation remission overtime.

It is interesting to note that En face OCT, OCT angiography and autofluorescence findings correlate well and are more severe in the left eye. Although the presence of an epiretinal membrane, a frequent finding in patients with posterior uveitis [12] and the fact that many studies have described FAF findings in uveitis involving the outer retina and choroid a predictor of poor central visual acuity, it was not what we found in our case.

We also agree with other authors [10] that it’s not possible to establish direct causal correlation between vaccination and uveitis, only presumable. But the increasing amount of reports with similar temporal correlations should draw our attention to a new spectrum of adverse reactions, especially in association to isolated YF vaccines.

Clinicians should be aware of clinical and particularly subclinical ocular manifestations such as subtle puntiform choriocapillaris changes as possible vaccine-related adverse events with potential to impact vision.

## Data Availability

Data is contained within the patient’s medical record and will not be distributed.

## Abbreviations

NR: normal range
OCT: optical coherence tomography
YF: yellow fever
